# Anatomorphometry of the brachial plexus under high-definition system: an experimental study in rats

**DOI:** 10.1590/acb370206

**Published:** 2022-05-02

**Authors:** Rui Sergio Monteiro de Barros, Deivid Ramos dos Santos, Renan Kleber Costa Teixeira, Nayara Pontes de Araújo, Danusa Neves Somensi, Atylla de Andrade Candido

**Affiliations:** 1 PhD, Associate Professor. Universidade do Estado do Pará – School of Medicine – Department of Experimental Surgery – Laboratory of Experimental Surgery – Belém (PA), Brazil.; 2 Fellow Master degree. Universidade do Estado do Pará – Postgraduate Program in Surgery and Experimental Research – Department of Microsurgery – Laboratory of Experimental Surgery – Belém (PA), Brazil.; 3 MD. Universidade do Estado do Pará – School of Medicine – Department of Experimental Surgery – Laboratory of Experimental Surgery – Belém (PA), Brazil.; 4 Graduate student. Universidade Federal do Pará – School of Medicine – Department of Experimental Surgery – Laboratory of Experimental Surgery – Belém (PA), Brazil.; 5 MD. Universidade do Estado do Pará – School of Medicine – Department of Neurology – Laboratory of Experimental Surgery – Belém (PA), Brazil.; 6 MD. Universidade do Estado do Pará –School of Medicine – Department of Experimental Hand Surgery – Laboratory of Experimental Surgery – Belém (PA), Brazil.

**Keywords:** Brachial Plexus, Nerve, Dissection, Anatomy

## Abstract

**Purpose:**

To study the anatomorphometry of the plexus brachialis (PB) of rats under a high-definition video system.

**Methods:**

Ten male Wistar rats discarded from other research that did not interfere in the morphology of the animal, respecting the principle of reduction, were used. All animals were submitted to the same protocol. Initially, the cervical region was shaved. The animals were placed in a dorsal position. A single elbow-to-elbow incision was performed and dissection started at the deltopectoral sulcus. The procedures were performed under a video system. To measure the structures, the Image J software was used.

**Results:**

All the PB evaluated originated from the C5-T1 spinal nerves. C5 and C6 converged to form the truncus superior, the root of C7 originated the truncus medius, and the confluence of C8 and T1 originated the truncus inferior. It was found the union of C7, C8, and T1 to form truncus inferomedialis instead of separate medial and inferior truncus. C8 (1.31 mm) was the thickest root, the truncus inferior (1.80 mm) and the nerve radialis (1.02 mm), were the thickest.

**Conclusions:**

The anatomy of the PB is comparable to humans, admitting variations. The videomagnification system is useful to perform microsurgical dissection.

## Introduction

Brachial plexus or plexus brachialis (PB) injuries in humans are very frequent and can cause devastating neurological damage, especially if hand function is affected. The main patients are young, causing a significant social and economic impact on society^1^^–^ 3.

The rat PB is considered a viable model in the area of microsurgical experimentation, especially due to its similarity to the human PB^4^^–^ 8 with structural and functional equivalence in several systems^4^^,^ 5. For this reason, it serves the purpose of researchers who seek explanations in the experimental field for relevant phenomena observed in humans^9^^,^ 10, mainly when new techniques are to be used to develop basic and advanced research, especially when studying the nerve under magnification^11^.

Despite the growing interest in the application of the rat as an experimental model, a certain scarcity of details regarding its anatomorphometry of PB and its collateral nerves was observed^6^–^8^. Therefore, the purpose of this work is to study the anatomy and morphometry of the PB of rats under a high-definition videomagnification system.

## Methods

The study followed the rules set out in the Brazilian national legislation on animal care (Law 11.794/08), which is based on NIH guidelines, and complied with the Council for International Organization of Medical Sciences ethical code for animal experimentation and the ARRIVE guidelines. The project was approved in advance by the Animal Use and Care Committee at the UEPA (protocol 02/19).

Ten male Wistar rats (20 weeks old), without diseases, were used in this study, weighing 400–450 g, discarded from other research that did not interfere in the morphology of the animal, respecting the principle of reduction^12^.

The rats were assigned according to the chronological order of the dissections, and the PB was designated by a number corresponding to the rat and a letter describing the laterality (R: right; L: left).

All animals were submitted to the same protocol. Initially, the cervical region was shaved. The animals were placed in a dorsal position with the forelimbs in 90° abduction. Under direct visualization, a single elbow-to-elbow incision was performed.

Dissection started at the deltopectoral sulcus. A proximal disinsertion of the deltoid’s clavicular portion was made first. Then, a disinsertion of the clavicular portion of the major pectoralis was made. A clavicle excision was made taking care not to damage the muscle subclavian and its innervation. The cleidomastoid muscle was resected as well as the anterior scalene for exposure of the PB.

A morphological description of the PB was made considering the segmentation of the spinal nerves until the formation of the collateral and terminal nerves. The nomenclature chosen followed the seventh edition of *Nomina Anatomica Veterinaria* 13.

The morphometric analysis was performed by obtaining the length (millimeters) measured from the lateral vertebrae body border identified during each dissection.

The procedures were performed under a video system composed by a high-definition Sony camcorder HDR-CX 150 set to 52× magnification, connected with a 4K 55-inch curved television, positioned in front of the surgeon, by a digital HDMI cable. Two white led light sources were used near the camera to provide adequate illumination of the operative field^11^^,^ 14.

During the video documentation, a millimeter paper was used as a reference for the measurements of the PB morphometric analysis. To measure the structures, the Image J software was used. This program allows the precise measurement of the structures, by the calculation of the distance between two points precisely. For all nerves, the largest diameter and the length were calculated.

BioEstat 5.4 software was used. The Student’s t-test was used to compare the study variables. Statistical significance was assumed at p < 0.05. All data were expressed as means ± standard deviation.

## Results

**Figure 1:**
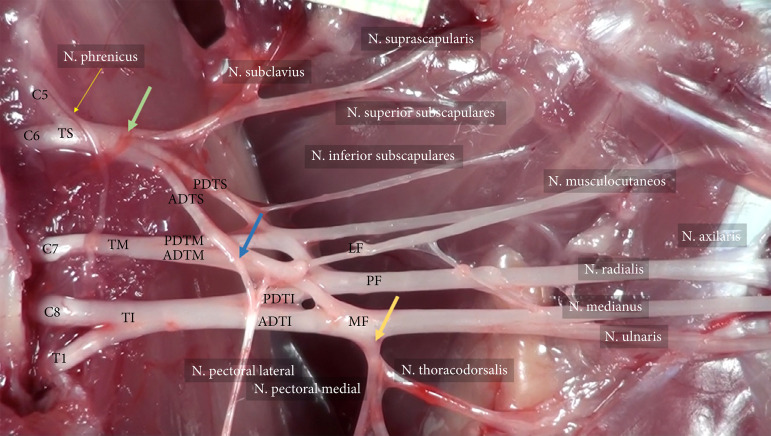
A common formation of plexus brachialis in rat. C5, C6, C7, C8, and T1: ventral roots of the PB; TS: truncus superior; TI: truncus inferior; TM: truncus medius; AD: anterior division; PD: posterior division; LF: lateral fascicle; MF: medial fascicle; PF: posterior fascicle; Green set-point: common origin of the subclavius, suprascapularis, and superior subscapulares nerve, from the TS; Blue set-point: lateral pectoral nerve origin point from the ADTS; Yellow set-point: the common origin of medial pectoral and nervus thoracodorsalis, from the MF.

All the PB evaluated originated from the C5 - T1 spinal nerves, with a small contribution from C4, which joined a branch of C5, giving rise to nerve dorsalis scapulae. Also, the nervus phrenicus originated from the C5 root in all animals (Fig. 1). There was no root contribution from T2 to T1 observed in this research.

In 90% of the cases, C5 and C6 converged to form the truncus superior (TS), the root of C7 originated the truncus medius (TM), and the confluence of C8 and T1, originated the truncus inferior (TI).

From TS emerged the anterior (ADTS) and posterior divisions (PDTS). The lateral fascicle was formed from ADTS, singly. The medial fascicle was formed from anterior divisions of TM and TI. The posterior divisions of TS, TM, and TI form the posterior fascicle (Fig. 1).

**Figure 2:**
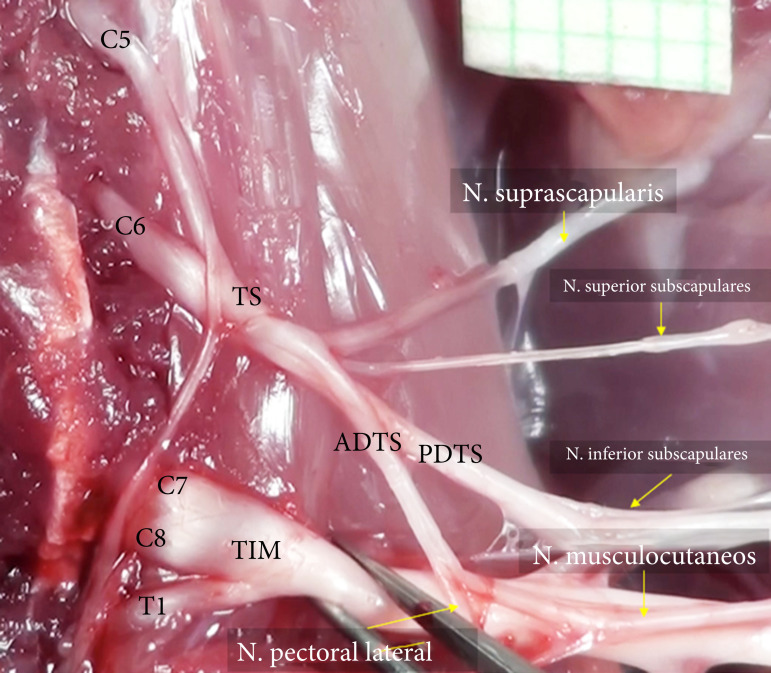
A variation of the truncus inferio-medialis of right PB in the rat. C5, C6, C7, C8, and T1: ventral roots of the PB, TS: truncus superior; TIM: truncus inferio-medialis. AD: anterior division; PD: posterior division.

The union of C7, C8, and T1 form truncus inferomedialis was encountered in two animals instead of separate medial and inferior truncus (Fig. 2). A common origin was found for the subclavian, suprascapular, and superior subscapular nerves from TS, despite several anatomical variations of the collateral nerves.

The inferior subscapular nerve originated from the PDTS, while the nervus thoracicus lateralis originated from ADTS. The medial pectoral and thoracodorsal nerves have a common origin in the medial fascicle (Fig. 1).

The formation of the terminal nerves followed a constant segmentation from fascicles: the nervus musculocutaneus from the lateral fascicle; the nervi medianus and ulnaris, from the medial fascicle; and the nervi radialis and axilaris, from the posterior fascicle.

The mean values and standard deviations of the length from the lateral border of the cervical vertebral body are presented in Table 1, and the values of the PB thickness are shown in Table 2. No significant difference in laterality was found between the distance and thickness measurements of the evaluated plexus structures (p > 0.05). On the other hand, it is important to emphasize that the average value of the structures of the rat PB reveals that C8 (1.31 mm) is the thickest root, the TI is the thickest truncus (1.80 mm), and the radial (1.02 mm), the thickest nerve.

**Table 1:** Distance of neural structures from the lateral border of the vertebral body (in mm) PB, 2022.

	**Media (mm) ± SD**		
	**Right**	**Left**	**Both**
The convergence point of C5 and C6 to originate TS	5.0 ± 1.24	4.76 ± 2.00	4.87 ± 1.62
The convergence point of C8 and T1 to originate TI	5.9 ± 1.57	5.91 ± 1.80	5.91 ± 1.64
From TS’s bifurcation point to AD and PD	7.3 ± 1.40	6.64 ± 1.45	6.99 ± 1.43
From TM’s bifurcation point to AD and PD	7.9 ± 1.40	8.66 ± 2.76	8.34 ± 2.23
From TI’s bifurcation point to AD and PD	13.0 ± 3.09	12.30 ± 2.85	12.64 ± 2.91
From C5-C4 convergence point to originate the NSD	3.7 ± 0.72	2.67 ± 1.15	3.20 ± 1.08
Origin of the nervus phrenicus, from C5	4.9 ± 0.93	3.85 ± 0.66	4.37 ± 0.95
Common origin of the nervi subclavius, suprascapularis, and superior subscapularis, from the TS.	7.6 ± 1.55	6.65 ± 1.06	7.13 ± 1.38
Inferior subscapular nerve origin point, from the PDTS	12.7 ± 3.37	12.72 ± 3.62	12.73 ± 3.41
Lateral pectoral nerve origin point from the ADTS	12.0 ± 2.42	10.72 ± 3.52	11.36 ± 3.01
Common origin of medial pectoral and nervus thoracodorsalis, from the MF	17.3 ± 2.41	15.84 ± 2.88	16.57 ± 2.69
Nervus musculocutaneus penetration point in the coracobrachialis muscle	27.4 ± 1.90	26.35 ± 3.89	26.84 ± 3.07
Nervus axilaris origin, from the PF	28.0 ± 3.37	23.42 ± 7.78	25.71 ± 6.29
Nervus radialis origin, after nervus axilaris origin	28.7 ± 4.53	27.88 ± 4.93	28.30 ± 4.63
Common origin of nervi medianus and ulnaris, after the of the nervi medial pectoral and thoracodorsalis	18.0 ± 3.38	16.72 ± 4.01	17.38 ± 3.67

The root of C7 cannot be distinguished from the truncus medius concerning distance from the vertebral body; TS: truncus superior; TM: truncusmedius; TI – truncus inferior; AD: anterior division; PD: posterior division; LF: lateral fascicle; PF: posterior fascicle; MF: medial fascicle.

**Table 2:** Plexus brachialis nerve thickness, 2022.

**Nerve structure**	**Media (mm) ± SD**		
	**Right**	**Left**	**Both**
C5	0.80 ± 0.08	0.72 ± 0.15	0.76 ± 0.13
C6	1.09 ± 0.19	1.00 ± 0.22	1.05 ± 0.21
C7	1.16 ± 0.24	1.13 ± 0.22	1.14 ± 0.23
C8	1.42 ± 0.44	1.20 ± 0.13	1.31 ± 0.34
T1	1.05 ± 0.18	0.93 ± 0.22	0.99 ± 0.21
TS	1.51 ± 0.16	1.61 ± 0.33	1.56 ± 0.27
TM	1.32 ± 0.20	1.25 ± 0.27	1.29 ± 0.25
TI	1.95 ± 0.45	1.64 ± 0.26	1.80 ± 0.40
Scapular dorsal nerve (from C5)	0.42 ± 0.12	0.47 ± 0.12	0.44 ± 0.12
Nervus phrenicus	0.34 ± 0.08	0.31 ± 0.06	0.33 ± 0.07
Nervus subclavius	0.35 ± 0.10	0.28 ± 0.10	0.32 ± 0.11
Nervus suprascapularis	0.52 ± 0.13	0.46 ± 0.06	0.50 ± 0.11
Superior subscapular nerve	0.34 ± 0.09	0.29 ± 0.07	0.32 ± 0.09
Inferior subscapular nerve	0.72 ± 0.12	0.58 ± 0.12	0.64 ± 0.14
Lateral pectoral nerve	0.42 ± 0.05	0.44 ± 0.08	0.43 ± 0.07
Nervus thoracodorsalis	0.73 ± 0.36	054 ± 0.26	0.64 ± 0.32
Medial pectoral nerve	0.54 ± 0.18	0.48 ± 0.14	0.52 ± 0.16
Nervus axilaris	0.73 ± 0.17	0.86 ± 0.16	0.80 ± 0.18
Nervus radialis	0.87 ± 0.22	1.17 ± 0.21	1.02 ± 0.26
Nervus musculocutaneus	0.56 ± 0.06	0.51 ± 0.14	0.54 ± 0.11
Nervus medianus	0.68 ± 0.10	0.80 ± 0.19	0.75 ± 0.16
Nervus ulnaris	0.76 ± 0.18	0.77 ± 0.20	0.76 ± 0.19

TS: Truncus superior; TM: Truncus medius; TI: Truncus inferior.

## Discussion

### The videomagnification system

The microscopy system chosen in this work has several advantages over magnified videomicrosurgery: greater comfort for surgeon and staff; better access to the surgical field; easy and portable mounting system^11^^,^ 14. Also, since everyone on the team can see the operative field exposed 62 times on high-definition TV, there is better communication and error prevention, making the surgery less time-consuming.

Another advantage, besides the purchase price of all the videomagnification equipment being less than US$ 2,500, the system allows the visualization and measurement of tiny structures in real-time and the storage of the video to be played back later to students in microsurgical training^14^, meeting the principle of reduction^12^.

The automatic focus function of the video system adopted allows free manipulation of the microsurgical structures without losing the operative view. It differs from the conventional microscope, which, for good visualization of the field, it is necessary to manually correct the microfocus, causing longer surgical time.

It is important to emphasize that the ventral access route to the PB is difficult and requires the skills of an experienced surgeon. When using videomagnification, the third dimension is lost, increasing the degree of difficulty in manipulating small structures, which does not occur in conventional microscopy.

### The rat brachial plexus

The anatomical and morphometric study of the PB of rats is little explored and lacks investigations to support better research in the experimental field. This contrasts with human anatomy, which has extensive studies on the normality variation related to the plexus^6^–^10^.

Considering the PB origin, the contribution of the ventral roots of C5-T1 was observed in all animals, in agreement with Angelica-Almeida *et al.* 6 experimental findings, but different from those of Özbağ *et al.* 7, in which the contribution of C4 may be present in 66% of cases.

No contribution of the ventral root from T2 to T1 was detected in the formation of the PB of the rats evaluated, similar to the case studies of Bertelli *et al.* 4 and Özbağ *et al.* 7, but different from the results Angelica-Almeida *et al.* 6.

The TS, formed from the ventral roots of C5 and C6, the TM from C7, and the TI from C8 and T1 were present in this arrangement in 90% of the cases, similar to the results of Özbağ *et al.* 7, and Bertelli *et al.* 4. In some cases, it was encountered that the union of C7, C8, and T1 formed truncus inferomedialis instead of separate medial and inferior truncus.

The segmentation of the rat PB into divisions and fascicles occurred similarly to the human PB^11^. The lateral fascicle was formed from the anterior division of the TS; the posterior fascicle, from the posterior divisions of the three trunci; and the medial fascicle, from the anterior divisions of the TM and TI.

In the literature, the work that came closest to this description was that of Bertelli^15^, except about the lateral fascicle, which was formed from the anterior division of the TS and TM. On the other hand, in the analyses of Angelica-Almeida *et al.* 6, there was no fascicle formation in the rat PB.

Among the collateral nerves of the plexus of the rats evaluated, the nervus phrenicus stands out, always present in our study, arising from the ventral root of C5, similar to results found by Mueller *et al*.^16^. The long thoracic nerve, described as a component of the rat’s PB in experimental studies^6^^,^ 16, was not found in this work.

It is known that in humans the long thoracic nerve emerges from the roots of C5 to C7^1^^,^ 2, which differs from what was found in our study since in the 10 animals dissected, this nerve was not identified, suggesting that there may be another origin and that should be revisited.

In complete agreement with Bertelli^15^, this study corroborated the same formation of the terminal nerves, as the musculocutaneus nerve is the terminal branch of the lateral fascicle, the axillary, and radial nerves of the posterior fascicle, and the median and ulnar nerves of the medial fascicle.

The human BP is divided into supra- and infraclavicular, different from that found in rats in this study, which have the BP infraclavicularly. Despite this, the great segmental similarity of the roots, trunks, divisions, and fascicles is remarkable when compared to human anatomy. This allows the use of the animal model by several researchers. However, it is noteworthy that in some aspects the PB in rats differs in terms of nerve functionality^5^^,^ 6.

A great example of this is the scarce or absent contribution of the ulnar nerve in the flexor muscles of the forepaw, being the median nerve responsible for this function, disagreeing with the studies in humans in which the ulnar nerve shares this function together with the median nerve. Thus, experimental studies that aim to evaluate the ulnar nerve and its impact on paw flexion may be biased^2^^,^ 4 ^,^ 15.

### Morphometry

No statistical difference was found in the measurements between thickness and distance of the PB structures between the right and left sides, as to mean and standard deviation (p > 0.05).

Özbağ *et al.* 7 performed a morphometric analysis of the PB, comparing measurements of the plexus with parameters of the forelimb muscles. Such parameters were not used by us because they are difficult to measure and unreliable.

The radial nerve was the terminal nerve with the greatest thickness, regardless of laterality, contrary to what was reported^6^, in which the median is considered the thickest nerve of the rat PB. The other structures were not subject to discussion because they did not present correlated studies in the literature.

One of the reasons for this may be the difficulty in measuring such small structures by conventional means since no data related to the collateral and terminal branches of the studied plexus were found for comparative purposes, especially when analyzing diameter and length. Thus, it is noteworthy that the videomagnification system used in this work allows the measurement of small structures of the PB not measured so far, serving also as a starting point for further research involving arteries, veins, and muscles.

As studies on the variation of normality are still scarce in the literature, more work is needed to delimit exactly what normality and what variation of normality are found^17^^,^ 18.

This research shows, besides the PB nerves, collateral branches in rich detail up to their respective muscle insertions, highlighting the importance of the magnification system used to register this data^19^.

## Conclusion

The anatomy of the PB is comparable to humans, admitting variations. The most common morphological pattern of the rat plexus brachialis was formed from C5 to T1 spinal nerves, with contribution from C4. There is no statistical difference between the thickness and distance of the structures of the rat’s PB compared to the contralateral side. The videomagnification system is useful to perform microsurgical dissection and can be applied in other areas.
